# Target trial emulation to evaluate the effect of immune-related adverse events on outcomes in metastatic urothelial cancer

**DOI:** 10.1007/s00262-024-03871-7

**Published:** 2024-12-21

**Authors:** Renate Pichler, Josef Fritz, Sarah Maier, Melanie R. Hassler, Johanna Krauter, David D`Andrea, Ekaterina Laukhtina, Kilian Gust, Keiichiro Mori, Karl H. Tully, Dora Niedersuess-Beke, Lea Korber, Jasmin Alija Spiegelberg, Thomas Bauernhofer, José D. Subiela, Roman Mayr, Andreas Kronbichler, Marco Moschini, Jeremy Teoh, Benjamin Pradere, Shahrokh F. Shariat, Hanno Ulmer, Laura S. Mertens, European Association of Urology–Young Academic Urologists (EAU-YAU): Urothelial Carcinoma Working Group

**Affiliations:** 1 Department of Urology, Comprehensive Cancer Center, Medical University of Innsbruck, Anichstraße 35, 6020 Innsbruck, Austria; 2 Department of Medical Statistics, Informatics and Health Economics, Medical University of Innsbruck, Innsbruck, Austria; 3 Department of Urology, Comprehensive Cancer Center, Medical University of Vienna, Vienna, Austria; 4 Department of Urology, The Jikei University School of Medicine, Tokyo, Japan; 5 Department of Urology and Neurourology, Marien Hospital Herne, Ruhr-University Bochum, Herne, Germany; 6 Klinik Ottakring, I. Medizinische Abteilung, Zentrum Für Onkologie, Hämatologie Und Palliativmedizin, Vienna, Austria; 7 Division of Oncology, Department of Internal Medicine, Medical University of Graz, Graz, Austria; 8 Department of Urology, Hospital Universitario Ramón y Cajal, IRYCIS, Universidad de Alcala, Madrid, Spain; 9 Department of Urology, St. Josef Medical Center, University of Regensburg, Regensburg, Germany; 10 Department of Internal Medicine IV, Nephrology and Hypertension, Medical University of Innsbruck, Innsbruck, Austria; 11 Department of Urology, IRCCS Ospedale San Raffaele and Vita-Salute San Raffaele University, Milan, Italy; 12 Department of Surgery, S.H. Ho Urology Centre, The Chinese University of Hong Kong, Hong Kong, China; 13 Department of Urology, La Croix du Sud Hospital, Quint Fonsegrives, France; 14 Department of Urology, Medical University of Vienna, Vienna, Austria; 15 Department of Urology, University of Texas Southwestern Medical Center, Dallas, TX USA; 16 Department of Urology, Weill Cornell Medical College, New York, NY USA; 17 Department of Urology, Second Faculty of Medicine, Charles University, Prague, Czechia; 18 Department of Surgical Oncology (Urology), Netherlands Cancer Institute, Amsterdam, The Netherlands

**Keywords:** Urothelial cancer, Metastatic, Immunotherapy, Immune checkpoint inhibitors, Adverse events, Target trial emulation, Immortal time bias

## Abstract

**Background:**

Immune checkpoint inhibitors (ICIs) are an important therapeutic pillar in metastatic urothelial carcinoma (mUC). The occurrence of immune-related adverse events (irAEs) appears to be associated with improved outcomes in observational studies. However, these associations are likely affected by immortal time bias and do not represent causal effects. The aim of this study was to assess the effect of irAEs on outcomes while correcting for immortal time bias, using target trial emulation (TTE).

**Methods:**

TTE was contrasted to adjusted naïve and time-updated Cox models. We performed a multi-institutional retrospective study involving mUC patients under ICI. The primary objective was to assess the impact of irAEs on progression-free survival (PFS) and overall survival (OS). Secondary endpoints included the influence of irAEs on objective response rates (ORRs) to ICI and the influence of systemic corticosteroids on outcomes.

**Results:**

Among 335 patients (median age: 69 yrs), 69.6% received ICI in the second line or further lines. During a median follow-up of 21.1 months, 122 (36.4%) patients developed irAEs of any grade (grade ≥ 3: 14.9%). Hazard ratios (HRs) for PFS ranged from 0.37 for naïve adjusted Cox model to 0.88 (95% confidence interval (CI), 0.59–1.30) with time-updated covariates, and from 0.41 to 1.10 (95% CI, 0.69–1.75) for OS. TTE accounting for immortal time bias yielded a HR of 1.02 (95% CI, 0.72–1.44) for PFS, and 0.90 (95% CI, 0.62–1.30) for OS. In contrast to the naïve Cox model (HR = 2.26, 95% CI 1.26–4.05), the presence of irAEs was no longer a predictive factor for improved ORR in time-updated Cox models (HR = 1.27, 95% CI 0.68–2.36) and TTE (HR = 1.43, 95% CI 0.89–2.29). In patients with irAEs, systemic corticosteroids did not negatively impact survival.

**Conclusion:**

Using TTE, we were able to show that the occurrence of irAEs is no longer associated with better survival or improved response rates to ICI in mUC patients, in contrast to the naïve analysis. These findings demonstrate that TTE is a suitable formal framework to avoid immortal time bias in studies with time-dependent non-interventional exposures.

**Graphical abstract:**

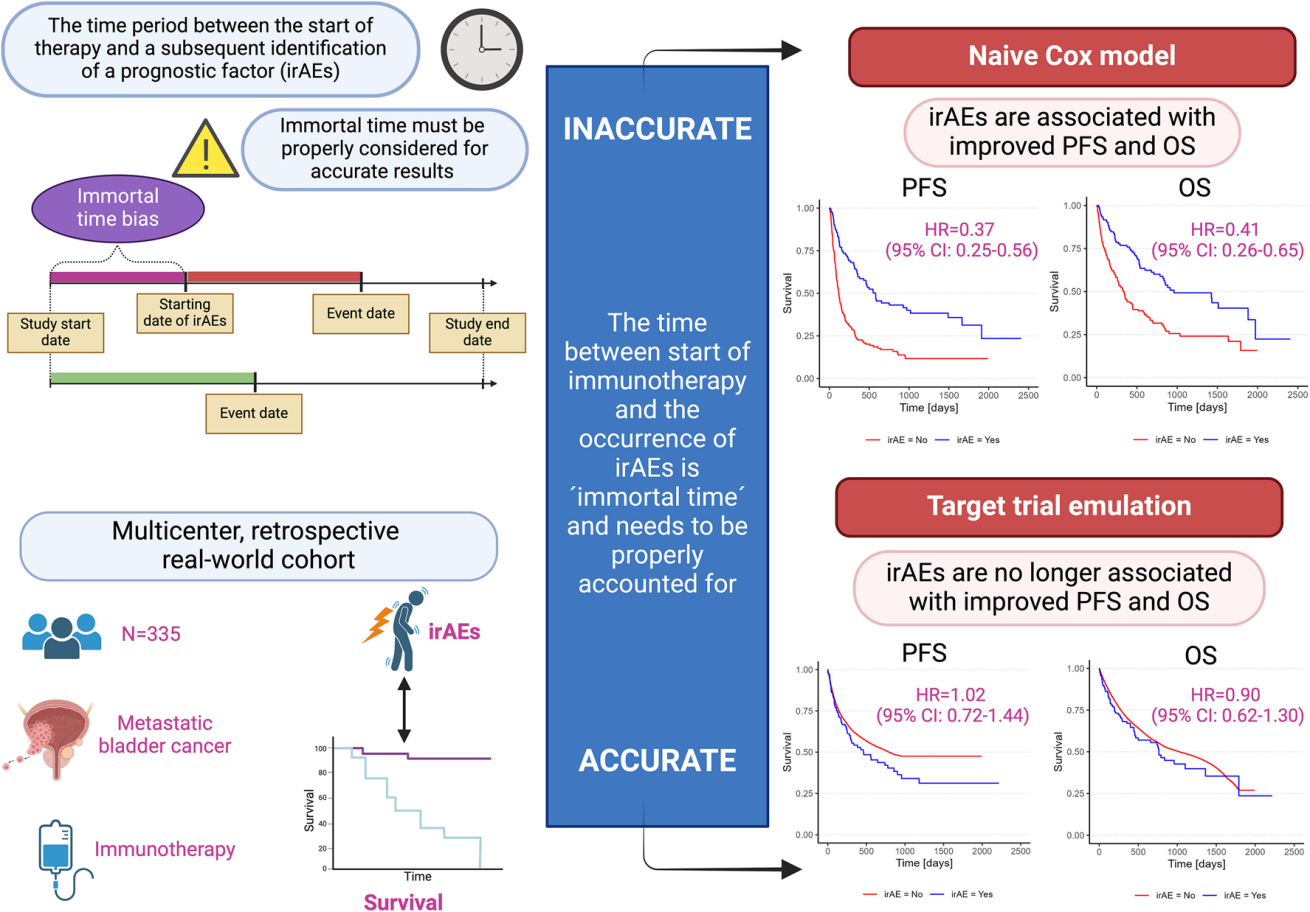

## Background

The introduction of immune checkpoint inhibitors (ICIs) has revolutionized the therapeutic landscape of metastatic urothelial cancer (mUC), previously limited to platinum-based chemotherapy alone, leading to significant improvements in outcomes [1, 2]. According to the current guidelines, ICI treatment is indicated (i) as first-line combination with enfortumab vedotin (EV), (ii) as first-line monotherapy in highly selective patients who are defined as EV- and platinum-ineligible with PD-L1 positive status, (iii) as second- or further-line treatment in patients who progressed during or after platinum-based combination chemotherapy, and (iv) as a switch maintenance strategy in patients achieving stable disease or better after platinum-based chemotherapy [3–5].

Immune-related adverse events (irAEs) are characterized by variable onset, presentation, severity, and outcomes [6, 7]. Across urological cancers, the pooled overall incidence for any-grade irAEs is 34.3%, with 10.2% experiencing grade ≥ 3 irAEs, depending on tumor entity, ICI agent, clinical settings, and therapy combinations [7]. The exact pathophysiology of irAEs associated with ICI is still unknown but is believed to be related to their important role in maintaining immunologic homeostasis [8]. Possible mechanisms underlying irAEs include enhanced T cell activity against antigens presented in tumors and healthy tissue, increased levels of inflammatory cytokines (interleukin-17) or preexisting autoantibodies, and elevated complement-mediated inflammation [8].

Several studies have suggested that patients experiencing irAEs tend to exhibit higher response rates and improved survival compared to those without such events [9]. Recently, data from the IMvigor210 and IMvigor211 trials demonstrated an inverse association between irAE occurrence and the risk of overall mortality, cancer-specific mortality, and disease progression in locally advanced or mUC patients treated with atezolizumab [10]. However, patients enrolled in clinical trials are highly selective, whereas real-world patients often present with more unfavorable characteristics [11]. Hence, real-world data are crucial for informing health policy decisions and validating clinical trial findings.

Despite the importance of real-world evidence, limited data are available on the paradoxical relationship between irAEs and outcomes in mUC patients [12, 13]. Furthermore, these data are prone to confounding and immortal time bias, which can influence the estimation of causal treatment effects. Although multivariable regression models have addressed these issues to some extent, additional statistical methods are necessary to improve the reproducibility and transparency of observational studies. Hernán et al. proposed the novel concept of target trial emulation (TTE) to address immortal time bias. TTE aims to replicate the design and conditions of a randomized study using observational data, allowing researchers to draw conclusions that better reflect causal relationships. This is achieved by “cloning” individuals at different starting points and applying weighting techniques to adjust the data, thereby producing more accurate causal estimates [14, 15].

In our study, we used the TTE approach, for the first time in this context, to evaluate the causal effect of irAEs on oncologic outcomes among mUC patients from a large real-world cohort.

## Methods

### Study design and patients

We conducted an observational retrospective analysis utilizing a multicenter YAU urothelial cancer collaboration group, comprising electronic medical records collected between January 2016 and January 2023. Data sharing was conducted in compliance with anonymization protocols as recommended by the General Data Protection Regulation [16], with the Young Academics Urologist Urothelial Carcinoma Working Group serving as the data repository entity. The study was approved by the local ethical committee of the Medical University of Innsbruck (study number AN2014-0121; 336/4.3).

Eligible patients included adult patients with either primary metastatic disease or those who underwent radical cystectomy (RC) for MIBC and subsequently developed metastatic disease during follow-up, and any consecutive systemic ICI therapy in the first line (atezolizumab, pembrolizumab), second line and further lines (pembrolizumab, nivolumab, atezolizumab) or as switch maintenance (avelumab). Patients with a diagnosis of mUC in the upper urinary tract, patients with locally advanced high-risk UC receiving adjuvant nivolumab after RC, those who were followed and treated elsewhere, and patients with incomplete data about outcomes and irAEs in the hospital`s computer database were excluded.

### Patient outcomes and follow-up investigations

Standard imaging was scheduled at baseline and then, every 3 to 4 months during ICI therapy. Imaging data were evaluated according to RECIST version 1.1 (complete response (CR), partial response, (PR) stable disease (SD), or progressive disease (PD)). Objective response rate (ORR) was defined as CR or PR from the time of ICI start to objectively documented disease progression or subsequent therapy, whichever occurred first. Patients received ICI therapy until unacceptable toxicity or radiographic progression. Each control visit involved a detailed medical history, complete laboratory blood examination, including thyroid levels, liver enzymes, and kidney values. Administration of systemic corticosteroids was depending on symptom grade of irAEs according to the current guidelines [17]. Dose interruptions and discontinuations, but not reductions, were permitted.

### Statistical analysis

The primary objective of the study was to assess the impact of irAEs on progression-free survival (PFS) and overall survival (OS). Secondary endpoints included ORR and the influence of systemic corticosteroids on outcomes. Since irAEs could occur anytime during follow-up, appropriate methodology is needed to avoid immortal time bias. As a reference analysis without considering immortal time bias, we estimated naïve adjusted Cox regression models defining irAE as exposure independent of the time point of its occurrence, because this approach has been frequently used previously [12, 13]. In these models, time zero where follow-up started was defined as the time of therapy initiation, and patients were followed up until the event of interest, death, or end of follow-up, whichever occurred first. In an attempt to obtain unbiased effect estimates of irAE, we used (i) time-updated Cox models, where all patients are initially assigned to the nonexposed group and transferred to the exposed group at the time point of irAE occurrence; and (ii) the TTE approach, a relatively new suggestion how to prevent immortal time bias in statistical analyses [15]. For the target trial emulation approach, we followed the methodology described elsewhere [18]. In brief, we emulate a sequence of 191 hypothetical clinical studies by using patients repeatedly (‘cloning’), starting each week of follow-up (‘time zero’). In each study, patients experiencing an irAE during the study’s first month were defined as exposed, patients with irAEs occurring later (or never) were defined as controls and the endpoint (OS/PFS/ORR/CRR) was censored at the irAE’s occurrence. To account for this artificial censoring, inverse-probability-of-censoring weighting was performed. In case of no irAE (and no artificial censoring), patients were followed up until the event of interest, death, or end of follow-up, as in the analyses above. Finally, the 191 emulated studies were analyzed in a pooled weighted Cox model, and 95% confidence intervals (CIs) were calculated using bootstrapping. All Cox models were adjusted for age, and sex, center, and therapy line, ECOG (0/ ≥ 1), and localization of metastases were added as strata. The TTE pooled weighted Cox model was additionally adjusted for study number as a natural cubic spline. All analyses were conducted in R, version 4.3.2 (R Foundation for Statistical Computing, Vienna, Austria), [19].

## Results

### Descriptive characteristics

Descriptive patient characteristics are presented in Table 1. Among 335 patients (74.6% male), the median age was 69 years (IQR, 59–76). Most patients (69.6%) received ICI in second-line or further lines, followed by first line (20.3%) and switch maintenance (10.1%). The median (IQR) follow-up was 21.1 (10.7–34.4) months.

**Table 1:** Patient characteristics, overall (*N* = 335) and stratified by irAE occurrence during follow-up

Characteristics		Total (*N* = 335)	irAEs during study follow-up		*p*-value^1^
			No (*N* = 213)	Yes (*N* = 122)	
Age [years], Median (IQR)		69.0 (59.0–76.0)	68.0 (59.0–75.0)	70.0 (60.0–77.0)	0.387
Sex, male		250 (74.6%)	168 (78.9%)	82 (67.2%)	0.026
ECOG					0.026
	0	171 (51.0%)	98 (46.0%)	73 (59.8%)	
	1	124 (37.0%)	90 (42.3%)	34 (27.9%)	
	> 1	40 (11.9%)	25 (11.7%)	15 (12.3%)	
Localization of metastases^1^					0.067
	Lymph node only disease	111 (34.6%)	63 (30.7%)	48 (41.4%)	
	Visceral metastases	210 (65.4%)	142 (69.3%)	68 (58.6%)	
	Liver metastases	71 (22.1%)	52 (25.4%)	19 (16.4%)	
IO-Agent					0.594
	Nivolumab	41 (12.2%)	29 (13.6%)	12 (9.8%)	
	Pembrolizumab	199 (59.4%)	126 (59.2%)	73 (59.8%))	
	Atezolizumab	52 (15.5%)	34 (16.0%)	18 (14.8%)	
	Avelumab	43 (12.8%)	24 (11.3%)	19 (15.6%)	
Therapy line					0.533
	First-line	68 (20.3%)	42 (19.7%)	26 (21.3%)	
	Second-line or further lines	233 (69.6%)	152 (71.4%)	81 (66.4%)	
	Switch Maintenance	34 (10.1%)	19 (8.9%)	15 (12.3%)	
Therapy duration [months], Median (IQR)		5.0 (2.0–12.0)	4.0 (2.0–8.5)	10.0 (4.0–26.0)	< 0.001
irAE grade, n (%)					–
	1–2	–	–	72 (59.0%)	
	3–4	–	–	45 (36.9%)	
	5	–	–	5 (4.1%)	
Systemic corticosteroid administration		–	–	62 (50.8%)	–
Deaths during FU		193 (57.6%)	137 (64.3%)	56 (45.9%)	0.001
Time until death [months], Median (IQR)^3^		6.0 (2.0–14.5)	5.2 (1.7–11.2)	12.9 (4.8–23.0)	< 0.001
Follow-up time [months], Median (IQR)^4^		21.1 (10.7–34.4)	16.8 (9.7–27.3)	27.9 (13.0–38.6)	0.014

^1^p-values from Wilcoxon test for quantitative data, and Fisher’s exact test for qualitative data

^2^Information missing for 14 patients

^3^Calculated based on the 193 patients who died during follow-up

^4^Calculated based on the 142 patients who did not die during follow-up

### Incidence of immune-related adverse events (irAEs)

**Fig. 1:**
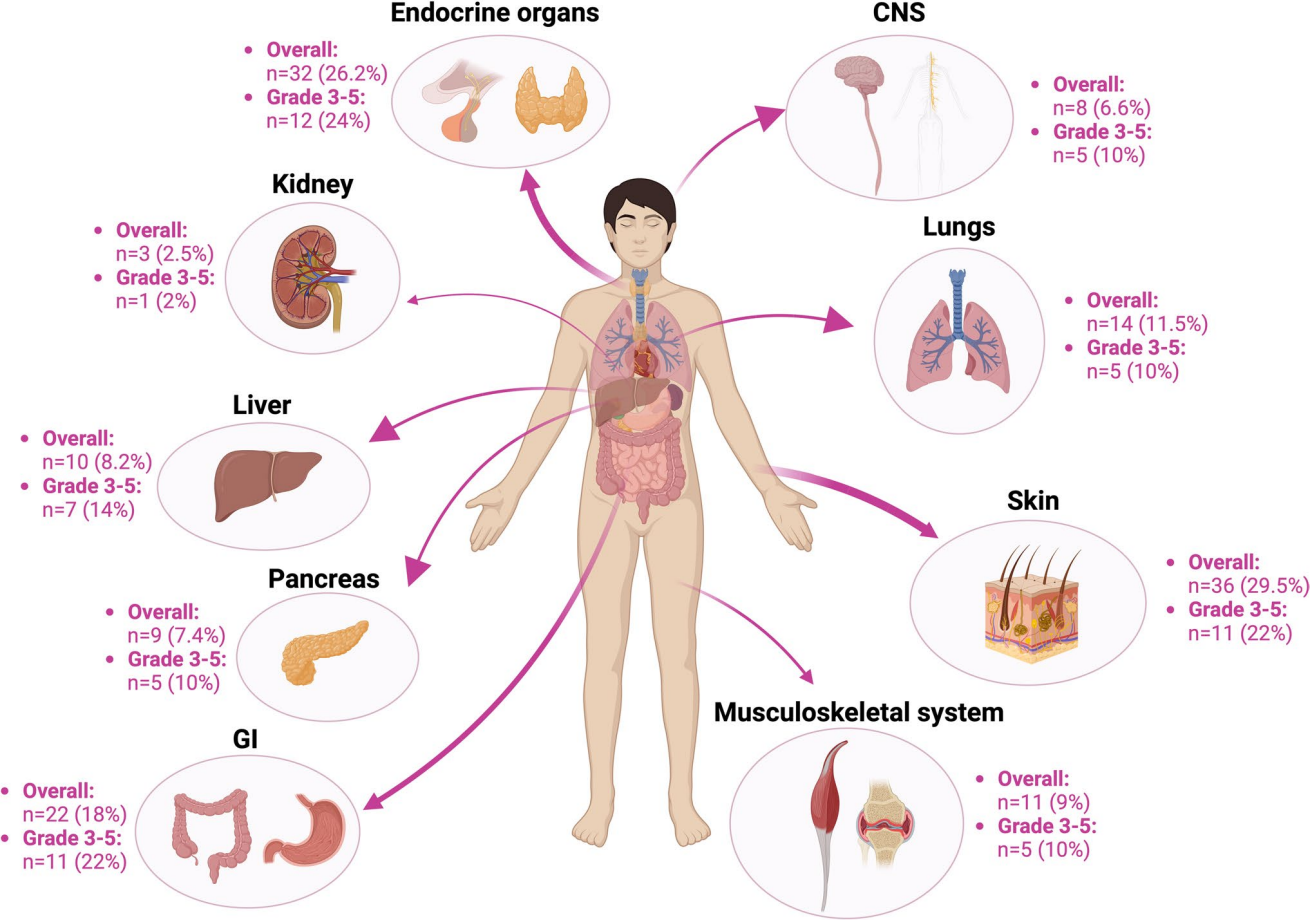
Overview of frequencies of immune-related adverse events (any grade and grade ≥ 3)

As shown in Table 1, irAEs occurred in 122 (36.4%) patients during study follow-up with a median onset of 3.0 (IQR, 1.4–7.6) months from the initiation of ICI treatment. Grade 3–5 irAEs occurred in 50 patients. irAEs were more frequent in women (*p* = 0.02) and less common in patients with an ECOG score ≥ 1 (*p* = 0.02). Patients with irAEs were treated with IO for a significantly longer time compared to those patients who did not exhibit irAEs (median 10 *vs.* 4 months, *p* < 0.001), because of their longer follow-up (median 28 *vs*. 17 months, *p* = 0.01) and survival time (median 13 *vs.* 5 months, *p* < 0.001), as shown in Table 1. The frequencies of irAEs (any grade and grade ≥ 3) according to the affected organ system are shown in Fig. 1.

### Immune-related adverse events (irAEs) and survival

**Fig. 2:**
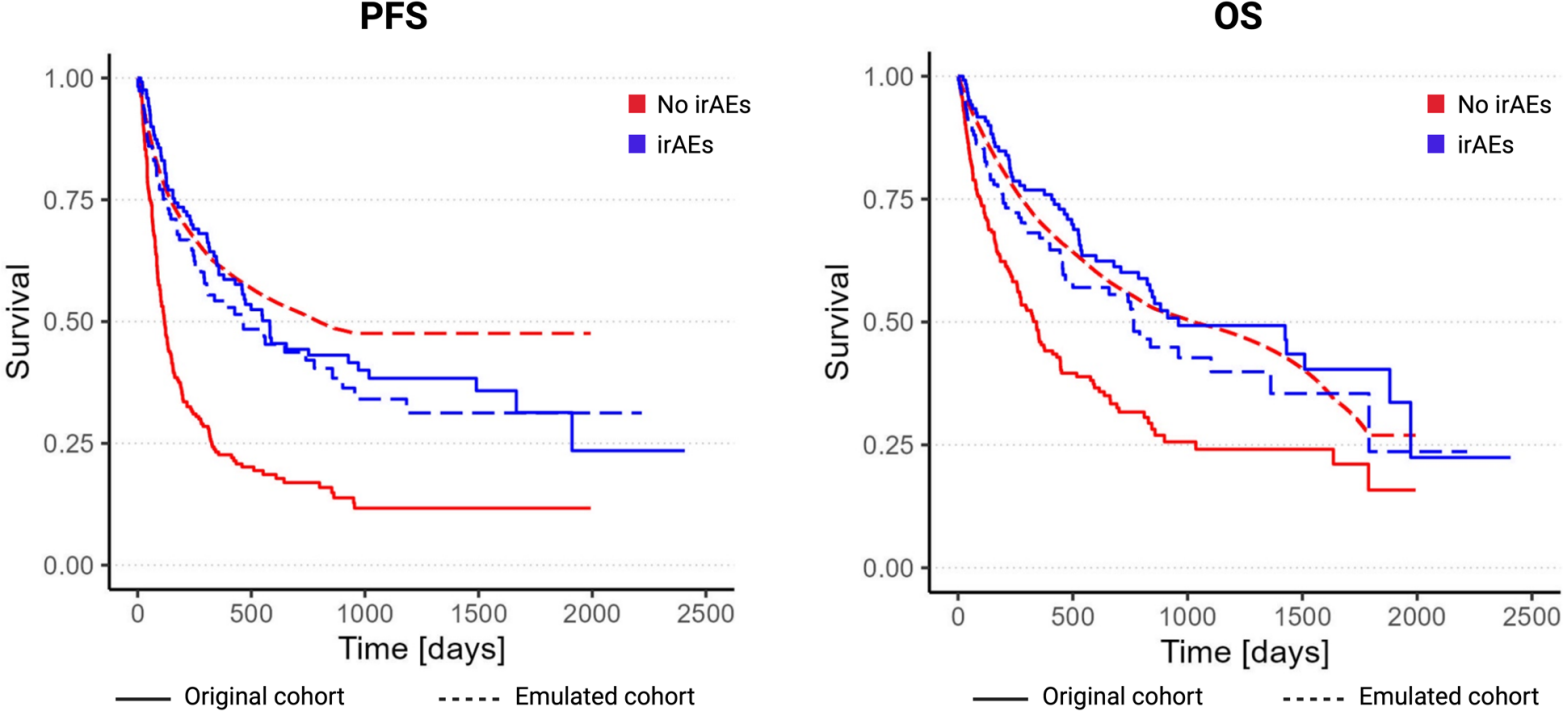
Contrasting Kaplan–Meier curves (irAEs *vs.* no irAEs) derived from the original cohort (naive analysis; solid lines) versus Kaplan–Meier curves from the emulated cohort (TTE approach; dashed lines). Red—no irAE group. Blue—irAE group. Numbers at risk taken from the naïve analysis. *(P* values by log-rank test. PFS: *p* < 0.001 for original cohort; *p* = 0.04 for emulated cohort; OS: *p* < 0.001 for original cohort; *p* = 0.2 for emulated cohort)

During follow-up, 239 (71.3%) patients confirmed PD and 193 (57.6%) died. The overall median PFS and OS was 6.2 and 17.2 months, respectively. Kaplan–Meier curves showed that PFS (*p* < 0.001) and OS (*p* < 0.001) was significantly improved in patients with irAEs when compared with patients without irAEs (Fig. 2), and multivariable naïve Cox models confirmed that the presence of irAEs was associated with improved outcomes (HR for PFS: 0.37, 95% CI 0.25–0.56; HR for OS: 0.41, 95% CI 0.26–0.65; Table 2). This paradoxical effect of irAEs on PFS and OS was irrespective of the therapy line (1L, 2L or maintenance; p_interactions_ > 0.1) and sex (p_interactions_ > 0.1). In the time-updated Cox models and the TTE approach, both PFS and OS did not show any statistically significant associations with the presence of irAEs anymore. HRs were 0.88, 95% CI 0.59–1.30 (time-updated Cox), and 1.02, 95% CI 0.72–1.44 (TTE) for PFS, and 1.10, 95% CI 0.69–1.75, and 0.90, 95% CI 0.62–1.30 for OS, respectively (Table 2). Kaplan–Meier curves on the emulated dataset confirmed these findings (p_log-rank test_ = 0.04 for PFS with better outcome in patients without irAEs; *p* = 0.2 for OS), Fig. 2.

**Table 2:** Hazard ratios (95% CIs) contrasting occurrence versus no occurrence of irAEs on survival and response rates to ICI using different estimation methods

	OS	PFS	ORR	CR
Original cohort, HR (95% CI)				
Naive cox	0.41 (0.26–0.65)	0.37 (0.25–0.56)	2.26 (1.26–4.05)	1.55 (0.68–3.51)
Time-updated cox	1.10 (0.69–1.75)	0.88 (0.59–1.30)	1.27 (0.68–2.36)	0.77 (0.35–1.70)
Emulated cohort, HR (95% CI)				
TTE	0.90 (0.62–1.30)	1.02 (0.72–1.44)	1.43 (0.89–2.29)	1.47 (0.78–2.76)

All models were for adjusted for age, and sex, study center, therapy line, ECOG (0/ ≥ 1), and localization of metastases (lymph node only disease/visceral metastases/missing) were added as strata. The TTE pooled weighted Cox model was additionally adjusted for study number as a natural cubic spline.

There were no statistically significant differences regarding sex or therapy line for any endpoint (all *p*-values for interaction > 0.1 for all four endpoints both for the naive and updated Cox models, and the Cox models from the emulated cohort)

OS, overall survival; PFS, progression-free survival; ORR, objective response rate; CR, complete response

### Immune-related adverse events (irAEs) and objective response (ORR) rate

The overall ORR was 32.7% (*n* = 108), with CR in 15.2% (*n* = 50). Patients who experienced irAEs had a significantly improved ORR in comparison to those that did not (ORR: 50.4% *vs.* 22.5%, *p* < 0.001; CR: 23.1% *vs.* 10.5%, *p* = 0.004). Multivariable analyses identified the presence of any irAEs as independent favorable prognostic factors for ORR using naïve Cox model (HR = 2.26, 95% CI 1.26–4.05). In time-updated Cox models (HR = 1.27, 95% CI 0.68–2.36) and TTE (HR = 1.43, 95% CI 0.89–2.29), the occurrence of irAEs was no longer a significant prognostic factor (Table 2).

### The impact of systemic corticosteroid administration on ORR and survival

**Fig. 3:**
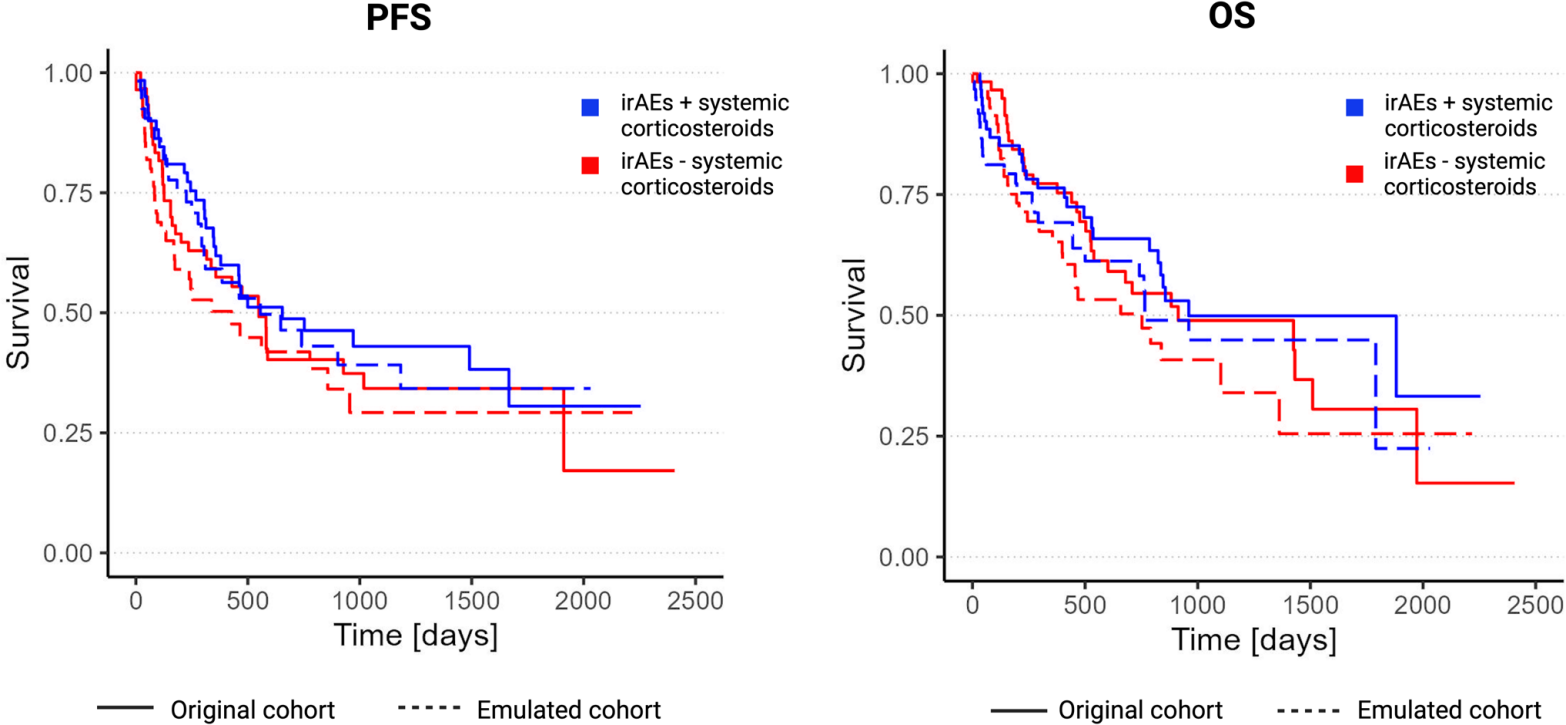
Contrasting Kaplan–Meier curves (systemic corticosteroids vs. no systemic corticosteroids) derived from the original cohort (naive analysis; solid lines) versus Kaplan–Meier curves from the emulated cohort (TTE approach; dashed lines). Red—irAE w/o systemic corticosteroids (irAE -). Blue—irAE with systemic corticosteroids (irAE +). Numbers at risk taken from the naïve analysis. *(P* values by log-rank test. PFS: *p* = 0.4 for original cohort; *p* = 0.2 for emulated cohort; OS: *p* = 0.6 for original cohort; *p* = 0.5 for emulated cohort)

Focusing on the irAEs subgroup (*n* = 122), survival curves showed no statistical differences between patients receiving systemic corticosteroids and lack thereof using naïve Cox model (PFS: HR = 0.41, 95% CI 0.17–0.99; OS: HR = 0.52, 95% CI 0.22–1.19), time-updated Cox models (PFS: HR = 0.51, 95% CI 0.24–1.08; OS: HR = 0.70, 95% CI 0.36–1.39), and TTE (PFS: HR = 0.84, 95% CI 0.51–1.41; OS: HR = 0.80, 95% CI 0.44–1.44), as shown in Fig. 3. On multivariable naïve Cox regression analysis, the presence of irAEs compared to no irAEs remained an independent favorable prognostic factor for survival irrespective of the administration of systemic corticosteroids. Using time-updated Cox models and the TTE approach, irAEs with or without systemic corticosteroids did again not show any significant effect on PFS and OS, as shown in Table 3.

**Table 3:** Hazard ratios (95% CIs) contrasting occurrence versus no occurrence of irAEs using different estimation methods, separately for irAEs treated with systemic corticosteroids, and irAEs not treated with systemic corticosteroids

	irAEs without systemic corticosteroid administration versus no AE		irAEs with systemic corticosteroid administration versus no AE		irAEs with *versus* without corticosteroid administration	
	OS	PFS	OS	PFS	OS	PFS
Original cohort, HR (95% CI)						
Naive cox	0.45 (0.27–0.77)	0.45 (0.28–0.73)	0.33 (0.17–0.67)	0.29 (0.14–0.57)	0.52 (0.22–1.19)	0.41 (0.17–0.99)
Time-updated cox	1.00 (0.59–1.67)	0.94 (0.60–1.49)	0.65 (0.31–1.37)	0.60 (0.31–1.16)	0.70 (0.36–1.39)	0.51 (0.24–1.08)
Emulated cohort, HR (95% CI)						
TTE	1.01 (0.65–1.56)	1.13 (0.73–1.75)	0.80 (0.48–1.33)	0.95 (0.62–1.46)	0.80 (0.44–1.44)	0.84 (0.51–1.41)

All models were for adjusted for age, and sex, center, therapy line, ECOG (0/ ≥ 1), and localization of metastases (lymph node only disease/visceral metastases/missing) were added as strata. The TTE pooled weighted Cox model was additionally adjusted for study number as a natural cubic spline

OS, overall survival; PFS, progression-free survival; ORR, objective response rate; CR, complete response

## Discussion

The prognosis and outcomes of patients with mUC have improved since the introduction of novel therapeutic targets such as ICI, antibody–drug conjugates (ADCs) and fibroblast growth factor receptor (FGFR) inhibitors with FGFR2 and FGFR3 alterations and fusions [3]. Focusing on ICI therapy in urothelial cancer, a meta-analysis showed that any irAEs occurred in 24.9% and grade ≥ 3 irAEs in 7.6% [7]. In our real-world study, the incidences of any irAEs were 36.4%, and grade ≥ 3 in 14.9%. Moreover, the presence of irAEs was irrespective of the used IO agent and therapy line. There is first evidence that irAEs are required to obtain a benefit from immune checkpoint inhibition [20]. In line with this finding, several studies have already reported that the occurrence of irAEs is associated with a better response to ICI and improved survival rates in mUC patients undergoing ICI therapy [10, 12, 13, 21, 22].

Nevertheless, it is imperative to acknowledge the presence of immortal time bias when exploring the association between the presence of irAEs and treatment response, given that irAEs can occur anytime during follow-up. Specifically, although the median onset of irAEs in our study was similar to other trials [8, 21] at 3 months, the temporal occurrence of irAEs varied strongly, ranging from 0 to 43 months. Thus, landmark analysis alone may not adequately address immortal time bias, as it may inadvertently exclude patients with survival periods shorter than 3 months [21].

Immortal time bias arises as a statistical distortion when there exists a period of time during which the outcome of interest cannot occur for certain patients. In our study context, this means that there are patients who do not survive long enough to develop irAEs, or their follow-up period includes a timeframe where irAEs cannot manifest [23]. Previous studies have addressed this bias using landmark analysis, time-updated analysis or extended Cox models [10, 12, 13, 21, 22].

The importance of considering causal inference for data interpretation is illustrated by the following example: when adjusted only for baseline covariates, a large pooled analysis including 1,747 mUC patients from seven ICI trials (5 trials enrolling patients with ICI after platinum-based therapy, 2 trials enrolling patients with ICI in the first-line) showed an improvement in OS among patients with related AEs of special interest (HR = 0.45; 95% CI 0.39–0.52) or irAEs (HR = 0.53; 95% CI 0.43–0.66) [24], thus being comparable to our study (HR = 0.41; 95% CI 0.26–0.58) performing only simple baseline adjustments.

To our knowledge, no prior study has employed TTE to eliminate immortal time bias in the causal investigation of the impact of irAEs on survival and response to ICI in mUC based on observational data. Specifically, TTE achieves this by designing a hypothetical “target trial” that would ideally answer the research question, allowing observational data to be analyzed as if collected in a randomized context. This method effectively reduces confounding, selection, immortal time, and other self-inflicted biases, which traditional multivariable regression models often fail to eliminate entirely. TTE strives to closely replicate a randomized study with the available data, providing causal estimates that align with those obtained from randomized study designs [14, 25, 26]. Consecutively, the emulation of a target trial using observational data will yield the same effect estimate as that of a RCT if the emulation is successful [14, 25, 26]. However, the requirements of TTE should be followed consequently: (i) specification of the target trial, definition of time zero and inclusion criteria with formulation of the causal question, (ii) cloning, and (iii) accounting for informative censoring by using inverse-probability-of-censoring weighting.

In our study, we could show for the first time in the context of mUC that TTE is a reproducible statistical method to avoid immortal time bias using real-world data. Focusing on the presence of irAEs and outcomes, the HRs for PFS significantly varied from 0.36 for simple baseline adjustments to 0.92 (95% CI 0.67–1.25) accounting for immortal time bias with time-updated covariates, and from 0.41 to 1.02 (95% CI 0.70–1.48) for OS. These data would result in a discordant interpretation and conclusion, emphasizing the importance of considering causal inference using observational data. TTE yielded a HR of 1.24 for PFS and 1.07 for OS, suggesting that the presence of irAEs no longer has any influence on survival in mUC patients. We were also able to show the same effect in the association between the presence of irAEs and response rates. In contrast to naïve Cox model (HR = 2.26), the presence of irAEs was no longer a favorable predictive factor in time-updated Cox models (HR = 1.27, 95% CI 0.68–2.36) and TTE (HR = 1.43, 95% CI 0.89–2.29).

A major limitation of our study is the small number of participants receiving ICI in the first-line setting (20.3%) or switch maintenance (10.1%), possibly affecting the study`s generalizability. Nevertheless, all Cox models in our analyses were adjusted not only for age, and sex, center, ECOG (0/ ≥ 1), but also for therapy line. However, it was not possible to adjust for irAE localization and the grade of irAEs, as these are characteristics that determine exposure. The predominance of male patients (74.6%) in our study is in line with the typical sex distribution in bladder cancer where male gender are four times more likely to develop bladder cancer than female gender [3]. However, a larger number of female patients would be necessary to further strengthen our hypothesis that the lack of influence of irAEs on outcomes was independent of gender, since gender may influence immune response and thus efficacy of ICI treatment (27, 28).

## Conclusion

Implementing target trial emulation for causal inference, we were able to show for the first time that there is no longer a significant association between the occurrence of irAEs (regardless of the administration of corticosteroids) and improved oncological outcomes and ICI response rates when adjusting the analysis to avoid immortal time bias. Therefore, common bias in observational studies can be limited using target trial emulation as a statistical methodology by conceptualizing them as attempts to hypothetical randomized trials answering causal questions of interest. Our data underline the importance of carefully designing observational studies based on real-world data, applying principles of randomized controlled trials to observational studies by target trial emulation. We believe these enhancements will provide readers with a clearer understanding of the TTE method, its advantages, and its significance in our research.

## Data Availability

No datasets were generated or analyzed during the current study.
